# Parents’ Use of Social Media for Health Information Before and After a Consultation With Health Care Professionals: Australian Cross-Sectional Study

**DOI:** 10.2196/48012

**Published:** 2023-10-20

**Authors:** Erika Frey, Catriona Bonfiglioli, Jane Frawley

**Affiliations:** 1 School of Public Health, Faculty of Health, University of Technology Sydney, Ultimo, New South Wales, Australia; 2 School of Communication, Faculty of Arts and Social Sciences, University of Technology Sydney, Ultimo, New South Wales, Australia

**Keywords:** social media, information seeking behavior, parenting, child, infant, health literacy, patient education, digital platform, information, health information, public health

## Abstract

**Background:**

Social media is a crucial source of health information for many parents due to its integration into modern life, raising critical concerns for public health. Parents use various social media platforms to find health information for their children, with most information created and shared by parents with no medical or health training. The extent to which parents seek health information from social media before and after a consultation and their motivations for doing so remain underresearched.

**Objective:**

This study aimed to investigate Australian parents’ use of social media for health information for their children, aged between 6 months and 5 years, before and after consulting with health care professionals.

**Methods:**

A representative cross-sectional survey of 1000 Australian parents with children aged 6 months to 5 years was conducted between November and December 2021. Data were cleaned and analyzed using IBM SPSS software. The primary outcomes were (1) parental motivation and prevalence of social media use for health information and (2) parental motivation for using social media before and after a consultation with their child’s health care professional.

**Results:**

Of the 1000 parents surveyed, 82.2% (n=822) reported using social media for health information for their child. Parents were more likely to consult social media before and after a health consultation if they were aged 30-39 or ≥50 years and born in Australia. Parents with higher levels of education were less likely to consult social media. Parents were motivated to seek health information before a consultation for a variety of reasons, including exchanging opinions and experiences (639/767, 83.3%), having information that is available 24/7 (622/767, 81.1%), receiving emotional support (599/767, 78.1%), having previous positive experiences (597/767, 77.8%), and having friends and family that use social media for health information (577/767, 75.2%). Parents sought information after a consultation to connect with parents with similar experiences (546/794, 68.8%), seek a second opinion (505/794, 63.6%), fact-check information provided by their health care professional (483/794, 60.8%), and look for other treatment options (353/794, 44.5%).

**Conclusions:**

Using social media for child health information is part of the modern parenting experience. It can be challenging to discern the quality of health information on social media, leaving parents open to incorrect information and misinformation. Although access to immediate social support is a welcomed feature of social media, receiving incorrect health information can have unwanted consequences for the child, family, health provider, and wider community. The upskilling of parental health literacy to navigate the unique health literacy challenges that social media brings, alongside the creation and delivery of accessible, evidence-based information in varying formats, is urgently required. The provision of this information is the responsibility of every level of the health system, not just the treating health care professional.

## Introduction

Social media platforms such as Facebook, Instagram, Twitter, Pinterest, and YouTube are key resources for parents seeking health information for their children [1-4]. The convenience and opportunity to meet like-minded parents has made social media central to modern parenting. In contrast to traditional health information accessed via books, internet web pages, or health care professionals, social media gives access to immediate health information from like-minded people, which is more likely to be ideologically aligned than evidence based. This democratization of health information risks downstream impacts including abstinence from formal health care [4], delay in seeking necessary health care [5], and the choice of non–evidence-based treatments [6], all of which impact health outcomes for children.

More broadly, the use of social media by parents has implications for health care professionals and public health. Although traditionally, health care professionals were one of the limited sources for health information that parents could find, health information can now be sourced almost instantaneously. This has direct impacts on how health care professionals provide care to their pediatric patients, with parents being able to actively seek out alternative information that may contradict or challenge the evidence-based information and treatment options that are being offered to them and their child during consultations [7,8]. Poor quality information from non–evidence-based sources has impacts on the community more broadly, with misinformation spread in the community setting by way of stories based on lived experience or rumor being exchanged between parents. Research has shown that misinformation can impact parents’ health decisions [9], for example, decisions about infectious disease and childhood immunization. Finally, when delayed evidence-based health care is eventually sought by parents, it is the health system that needs to provide it. This may result in more intense and resource-heavy care for the child [10].

Parents need to have a more diverse and honed set of health literacy skills when using social media for health information than previously required. This is due to the available health information being authored, compiled, or shared by parents that have little to no health expertise, making it almost certain to have a subjective bias to some degree. However, it is also because of this very fact that parents are seeking health information on social media—to gain insights from the lived experience of other parents further ahead on the same health journey that they have found themselves on. The skills needed to navigate social media health information sources include being able to discern quality evidence-based information from that of opinion [1] and politically or emotionally driven information [6]; tracing information to its source to determine context and relevance [11]; having the numeracy skills to be able to discern relative and absolute risk [9]; as well as being able to manage the sheer amount of information that is available [12], all of which is vying for the parent’s attention. In addition, parents need to have sophisticated social skills to be able to access some forms of health information, especially that of lived experience from other parents, where potentially complex interpersonal and group dynamics [13] can complicate access.

A variety of intrinsic and extrinsic factors have been found to motivate parents’ use of social media for health information. Intrinsic motivations include an increased sense of empowerment [6,14], self-efficacy, and self-determination and feeling more educated about the condition of concern [15,16]. Extrinsic motivators include being able to socialize [17] with like-minded people [18] to exchange support and advice [14,17,19], being offered reassurance and validation for health decisions [20], normalizing the challenges experienced [21], and having a sense of safety and privacy [18].

Three studies to date have provided evidence that parents use social media for health information before and after a diagnosis because they are medically underserved [14], want to use alternative health care [6], lack information that aligns with their health goals for their child [18], or lack appropriate information from health care professionals [18]. These studies focus on niche groups (those who are vaccine hesitant, infants with severe combined immunodeficiency, and mothers who exclusively express and bottle-feed) [4,14,18]. Social media’s utility has been demonstrated within these niche groups. However, considering social media’s ubiquity and fast pace of adoption and integration into people’s lives on a population level, we sought to understand how prevalent parents’ use of social media for health information before and after a health consultation is, alongside the reasons that parents use social media for health information. This representative study investigated the use of social media for health information among Australian parents, before and after consulting with health care professionals.

## Methods

### Study Design

A national quantitative cross-sectional survey was conducted between November and December 2021 among Australian parents of children aged 6 months to 5 years. For this study, “parent” was defined as anyone that was a biological parent, adoptive parent, or court-appointed guardian or caregiver of a child aged between 6 months and 5 years.

### Variables

A 47-item survey developed from previously validated tools combined multiple-choice questions with optional open-text fields and Likert scales [22,23]. The first section explored parents’ use of social media, the information sought, and motivations for using social media for child health information. The next section asked parents about their motivations for using social media before consulting with a health care professional. The final section asked parents their motivations for using social media for health information after consulting with a health care professional. Demographic data were collected.

A web-based research company (Quality Online Research [QOR]) was engaged to recruit parents from their web-based panel of preregistered participants and to administer the survey. Parents were recruited by way of a single-use email link, preventing multiple responses from a single participant. Eligibility criteria included being an Australian citizen or permanent resident who is caring for a child aged 6 months to 5 years. The company identified the participants by the demographic information that the participants provided when they joined the panel in preparation for survey opportunities. For our study, this was guided by our inclusion criteria. Exclusion criteria were limited command of the English language and not having an active social media account.

Due to the large and unknown population of parents of children aged 6 months to 5 years, a sample size of 1000 was chosen to give a CI of 3.1. Stratification parameters of the Australian 2016 census [24] ensured that the sample was representative of Australian states and territories and gender demographics. Parents were offered a small incentive (about Aus $2.80 [US $1.80]) to participate. The survey was refined with 2 rounds of corrections during the prepilot phase to ensure skip steps and question formatting were done correctly. The survey was pilot-tested among 122 parents, with responses checked by researchers for quality before being formally launched in the field. Fieldwork took 16 days (November to December 2021) to gather a total of 1000 eligible completed surveys (including the pilot test) from parents. The survey was kept at arm’s length from the researchers and was administered by QOR, with the data being cleaned and anonymized by QOR before being transferred to the researchers for analysis. The cleaned data were checked for quality control by researchers before commencing statistical analysis.

### Ethical Considerations

Participants were presented with a participant information sheet (PIS) as the first screen after opening the email link from QOR. This PIS informed participants about the project, why they have been invited (inclusion criteria), what their participation will involve, and the risks and inconvenience they can expect. They were also made aware that their participation was entirely voluntary and that there would be no penalty for their withdrawal at any point during the survey. Participants indicated their consent by commencing the survey after reading the PIS. As the data were being collected by a third-party research company, the researchers were not, at any point, exposed to any identifying information. Participants were given direct contact information for both the Ethics Secretariat and the lead investigator, if they required further information or follow-up. There was a consent form that outlined the main points highlighted in the PIS, which participants were able to click out of if they wished to discontinue or click forward to continue with the survey. Discontinuing their participation in the survey (returning an incomplete survey) was counted as a withdrawal, with the data being excluded from the final analysis. Ethics approval was obtained from the Human Research Ethics Committee at the University of Technology Sydney (UTS HREC ETH21-6598). This report is guided by the STROBE (Strengthening the Reporting of Observational Studies in Epidemiology) statement [25] for cross-sectional studies with supplementary guidance from the Consensus-Based Checklist for Reporting of Survey Studies [26].

### Analysis

Data were imported to IBM SPSS Statistics for Mac (version 28; IBM Corp) [27] for analysis. Descriptive statistics were calculated for sociodemographic data and parental use of social media. *Χ*^2^ tests of association were conducted to determine which aspects of parent motivation were statistically significant. Logistic regression was used to determine the significant predictors of social media use for health information before or after a consultation with a health care professional. Covariates with *P*<.25 were entered into the model, as well as Socio-Economic Indexes for Areas (The Index of Relative Socio-economic Advantage and Disadvantage) [28] codes corresponding to postcodes. Statistical significance was set at *P*<.05 to produce the most parsimonious model. There were some missing data (26/1026, 2.5%), possibly either due to participant error when entering their postcode or circumventing the requirement to answer before proceeding to the next question.

## Results

Of the 1563 parents who opened the survey link in QOR’s email invitation, 1026 (65.6%) completed the survey. In all, 26 surveys were deemed ineligible for analysis upon further data cleaning, leaving 1000 surveys for analysis, indicating a 64% (1000/1563) completion rate. Of the 1000 Australian parents surveyed, 57.5% (n=575) identified as female, 41.3% (n=413) identified as male, 0.8% (n=8) identified as nonbinary, and 0.4% (n=4) preferred not to say. Only 9.4% (n=94) of parents were not born in Australia (Table 1). Australian-born participants were found to be statistically more likely to use social media for their children’s health than non–Australian-born participants (*P*=.009). Variations by gender (*P*=.59); marriage status (*P*=.64); location by state (*P*=.71); language spoken at home (*P*=.69); and metro, rural, or remote location (*P*=.50) were not statistically significant. Covariates with *P*<.25 were imported into a logistic regression for further analysis.

**Table 1.** Participant characteristics.

Characteristics		Total (n=1000), n (%)	Use social media (n=822), n (%)^a^	Do not use social media (n=178), n (%)^a^	*P* value
**Gender**					.59
	Male	413 (41.3)	340 (82.3)	73 (17.7)	
	Female	575 (57.5)	471 (81.9)	104 (18.1)	
	Nonbinary	8 (0.8)	8 (100)	0 (0)	
	Prefer not to say	4 (0.4)	3 (75)	1 (25)	
**Age group (years)**					.12
	18-29	308 (30.8)	255 (82.8)	53 (17.2)	
	30-39	412 (41.2)	343 (83.3)	69 (16.7)	
	40-49	235 (23.5)	193 (82.1)	42 (17.9)	
	≥50	45 (4.5)	31 (68.9)	14 (31.1)	
**Location (by state or territory)**					.71
	New South Wales	322 (32.2)	268 (83.2)	54 (16.8)	
	Australian Capital Territory	13 (1.3)	10 (76.9)	3 (23.1)	
	Queensland	205 (20.5)	174 (84.9)	31 (15.1)	
	Victoria	269 (26.9)	214 (79.6)	55 (20.4)	
	South Australia	75 (7.5)	59 (78.7)	16 (21.3)	
	Tasmania	29 (2.9)	25 (86.2)	4 (13.8)	
	Northern Territory	4 (0.4)	4 (100)	0 (0)	
	Western Australia	83 (8.3)	68 (81.9)	15 (18.1)	
**Education**					.05
	High school	273 (27.3)	219 (80.2)	54 (19.8)	
	Trade qualification^b^	193 (19.3)	150 (77.7)	43 (22.3)	
	University qualification	534 (53.4)	453 (84.8)	81 (15.2)	
**Marital status**					.64
	Never married	207 (20.7)	169 (81.6)	38 (18.4)	
	Married or de facto marriage	754 (75.4)	623 (82.6)	131 (17.4)	
	Separated, divorced, or widowed	39 (3.9)	30 (76.9)	9 (23.1)	
**Country of birth**					.009
	Australia	906 (90.6)	754 (83.2)	152 (16.8)	
	Outside of Australia	94 (9.4)	68 (72.3)	26 (27.7)	
**Language spoken at home**					.69
	English	955 (95.5)	786 (82.3)	169 (17.7)	
	Other	45 (4.5)	36 (80)	9 (20)	
**SEIFA^c^ (n=974 valid responses)**					.18
	Q1 (highest)	213 (21.3)	179 (84)	34 (16)	
	Q2	218 (21.8)	178 (81.7)	40 (18.3)	
	Q3	217 (21.7)	168 (77.4)	49 (22.6)	
	Q4 (lowest)	326 (32.6)	275 (84.4)	51 (15.6)	
First child		769 (76.9)	623 (81)	146 (19)	.07
Metro		601 (60.1)	498 (82.9)	103 (17.1)	.50

a Percentages reflect the proportion of each subgroup (ie, the denominator is the n value in the Total column).

b Apprenticeship or other training to become a tradesperson.

c SEIFA: Socio-Economic Indexes for Areas (The Index of Relative Socio-economic Advantage and Disadvantage) [28].

A majority (822/1000, 82.2%) of parents used social media for information about their child’s general health and well-being. Parents were asked about their general motivations for using social media for health information (Table 2), before and after consulting a health care professional. Health care consultations were not defined beyond “visited the health care professional of your choice” to include any clinic or hospital visit. Parents’ use of social media for health information before a consultation was motivated by the ability to exchange opinions and experiences with other parents (639/767, 83.3%; *P*=.002), the information being available 24/7 (622/767, 81.1%; *P*<.001), receiving emotional support from other parents (599/767, 78.1%; *P*=.002), positive previous experiences using social media for health information (597/767, 77.8%; *P*<.001), having friends and family use social media for health information (577/767, 75.2%; *P*<.001), and the information being up to date (518/767, 67.5%; *P*<.001). Parents’ motivations for using social media after a consultation were similar, with the addition of anonymity while seeking health information (543/749, 72.5%; *P*=.009).

**Table 2.** Australian parents’ sentiments about using social media for health information.

Sentiment		Total (n=822), n (%)^a^	Use social media before an HCP^b^ visit, n (%)^a^		*P* value	Use social media after an HCP visit, n (%)^a^		*P* value
			Yes (n=767)	No (n=55)		Yes (n=749)	No (n=73)	
**The information is available 24/7**					<.001			.008
	Agree	652 (79.3)	622 (81.1)	30 (54.5)		598 (79.8)	54 (74)	
	Neutral	113 (13.7)	96 (12.5)	17 (30.9)		98 (13.1)	15 (20.5)	
	Disagree	57 (6.9)	49 (6.4)	8 (14.5)		53 (7.1)	4 (5.5)	
**The information is up to date**					<.001			<.001
	Agree	540 (65.7)	518 (67.5)	22 (40)		512 (68.4)	28 (38.4)	
	Neutral	213 (25.9)	189 (24.6)	24 (43.6)		174 (23.2)	39 (53.4)	
	Disagree	69 (8.4)	60 (7.8)	9 (16.4)		63 (8.4)	6 (8.2)	
**I can retain my anonymity (people don’t know who I am)**					.10			.009
	Agree	583 (70.9)	551 (71.8)	32 (58.2)		543 (72.5)	40 (54.8)	
	Neutral	168 (20.4)	150 (19.6)	18 (32.7)		142 (19)	26 (35.6)	
	Disagree	71 (8.6)	66 (8.6)	5 (9.1)		64 (8.5)	7 (9.6)	
**I have had good experiences with it**					<.001			<.001
	Agree	624 (75.9)	597 (77.8)	27 (49.1)		583 (77.8)	41 (56.2)	
	Neutral	168 (20.4)	142 (18.5)	26 (47.3)		136 (18.2)	32 (43.8)	
	Disagree	30 (3.6)	28 (3.7)	2 (3.6)		30 (4)	0 (0)	
**My friends and family use them as well**					<.001			<.001
	Agree	603 (73.4)	577 (75.2)	26 (47.3)		565 (75.4)	38 (52.1)	
	Neutral	160 (19.5)	136 (17.7)	24 (43.6)		135 (18)	25 (34.2)	
	Disagree	59 (7.2)	54 (7)	5 (9.1)		49 (6.5)	10 (13.7)	
**It’s a place where I can exchange opinions and experiences with other parents**					.002			<.001
	Agree	678 (82.5)	639 (83.3)	39 (70.9)		624 (83.3)	54 (74)	
	Neutral	119 (14.5)	106 (13.8)	13 (23.6)		105 (14)	14 (19.2)	
	Disagree	25 (3)	22 (2.9)	3 (5.5)		20 (2.7)	5 (6.8)	
**To receive emotional support from other parents**					.002			.007
	Agree	633 (77)	599 (78.1)	34 (61.8)		584 (78)	49 (67.1)	
	Neutral	142 (17.3)	122 (15.9)	20 (36.4)		122 (16.3)	20 (27.4)	
	Disagree	47 (5.7)	46 (6)	1 (1.8)		43 (5.7)	4 (5.5)	

a Some percentages may not sum to 100% due to rounding.

b HCP: health care professional.

When asked which statements were true of their use of social media for health information, parents’ responses varied (Table 3). A total of 60% (503/838) of parents sought general information about a condition of concern for their child on social media. Parents used social media to determine if medical attention was required (363/838, 43.3%) and seek information about alternative treatments such as natural remedies (350/838, 41.8%) and other medical treatments (293/838, 35%) for the condition of concern. When seeking general health information, parents were the least likely to use social media for information about self-management strategies (292/838, 34.8%).

**Table 3.** Parental motivations for using social media for children’s health information.

Motivations for using social media		Yes^a^, n (%)
**Children’s health information in general (n=838)**		
	To seek general information about the health problem or illness	503 (60)
	To determine if medical attention was required	363 (43.3)
	To seek information about alternative treatments for the health problem or illness	350 (41.8)
	To seek information about possible medical treatments for the health problem or illness	293 (35)
	To seek information about self-management strategies	292 (34.8)
**Health information *before* a health care professional visit (n=823)**		
	To seek general information about the health problem or illness	510 (62)
	To determine if medical attention was required	425 (51.6)
	To seek information about alternative treatments for the health problem or illness	351 (42.6)
	To seek information about possible medical treatments for the health problem or illness	326 (39.6)
	To seek information about medications	325 (39.5)
**Health information *after* a health care professional visit (n=794)**		
	To find examples of lived experience	546 (68.8)
	I wanted a second opinion	505 (63.6)
	To check the information I received at the doctor’s office	483 (60.8)
	To seek further information about the health problem or illness	453 (57.1)
	To determine if further medical attention was required	373 (47)
	I did not receive enough information at the doctor’s office or clinic	364 (45.8)
	The information from the doctor’s office was unclear	357 (45)
	To seek information about alternative treatments for the health problem or illness	353 (44.5)
	To seek information about possible medical treatments for the health problem or illness	314 (39.5)
	To seek information about medications	291 (36.6)

a Parents were asked to check all that applied.

When parents were asked about seeking information on social media before a consultation, most (510/823, 62%) looked for information about the health condition. About half (425/823, 51.6%) sought to determine if medical attention was required. Alternative treatments (351/823, 42.6%) were sought also, with 39.5% (326/823) of parents seeking information about (other) possible medical treatments.

When parents were asked about their motivations for using social media for health information after visiting a health care professional, 68.8% (546/794) stated they did so because they wanted to find examples of lived experience. Parents also wanted a second opinion (505/794, 63.6%), to check the information provided during the consultation (483/794, 60.8%), or to seek further information about the health condition (453/794, 57.1%). Just under half of all parents who used social media after a consultation did so to determine if further medical attention was required (373/794, 47%), having felt that they did not receive enough information from their health care professional (364/794, 45.8%), or that the information they were given was unclear (357/794, 45%). Other reasons included wanting to seek alternative treatments (353/794, 44.5%), information about possible medical treatments for the condition (314/794, 39.5%), or information about medications (291/794, 36.6%).

The results of the logistic regression conducted (Table 4) show that Australian-born parents were more likely to use social media for health information for their children both before (odds ratio [OR] 2.545, 95% CI 1.521-4.259) and after a health consultation (OR 2.045, 95% CI 1.228-3.407) than those born outside of Australia. Parents aged 30-39 years were the most likely to use social media before (OR 3.212, 95% CI 1.475-6.996) and after a consultation (OR 3.799, 95% CI 1.821-7.926) when compared to the reference group of parents aged 18-29 years. Parents aged ≥50 years were also more likely to use social media before (OR 2.324, 95% CI 1.066-5.068) and after a consultation (OR 3.428, 95% CI 1.625-7.233) than parents aged 18-29 years.

Education was a significant predictor for social media use among parents before and after a consultation. Parents with university (OR 0.513, 95% CI 0.332-0.794) or trade qualifications (OR 0.535, 95% CI 0.352-0.814) were less likely to consult social media before a consultation than parents with high school qualifications. Parents with a university (OR 0.515, 95% CI 0.319-0.719) or trade qualification (OR 0.631, 95% CI 0.395-0.882) were also less likely to use social media for health information after a health consultation.

**Table 4.** Predictors for parental use of social media before and after a consultation with a health care professional.

Predictor		Use social media before consultation			Use social media after consultation		
		OR^a^ (95% CI)		*P* value	OR (95% CI)		*P* value
**SEIFA** ^b^ ^,^ ^c^							
	Q1 (highest)	Reference			Reference		
	Q2	1.666 (1.027-2.700)		.04	1.270 (0.796-2.027)		.32
	Q3	0.795 (0.519-1.335)		.45	0.685 (0.429-1.093)		.11
	Q4 (lowest)	1.252 (0.776-2.086)		.34	0.885 (0.551-1.423)		.62
**Age group (years)**							
	18-29	Reference			Reference		
	30-39	3.212 (1.475-6.996)		.003	3.799 (1.821-7.926)		<.001
	40-49	1.918 (0.917-4.010)		.08	1.818 (0.912-3.626)		.09
	≥50	2.324 (1.066-5.068)		.03	3.428 (1.625-7.233)		<.001
**Country of birth**							
	Outside of Australia	Reference			Reference		
	Australia	2.545 (1.521-4.259)		<.001	2.045 (1.228-3.407)		.006
**Education**							
	High school	Reference			Reference		
	Trade qualification	0.535 (0.352-0.814)		.003	0.631 (0.395-0.882)		.01
	University qualification	0.513 (0.332-0.794)		.003	0.515 (0.319-0.719)		<.001

a OR: odds ratio.

b SEIFA: Socio-Economic Indexes for Areas (The Index of Relative Socio-economic Advantage and Disadvantage) [28].

c 974 responses included.

## Discussion

### Principal Findings

The Australian parents most likely to use social media for health information before and after a consultation were aged 30-39 years (Generation Y or millennials) and born in Australia. Reasons for this could include that because Generation Y or millennials, as digital natives, have their parenting experience colored by their everyday use of social media, digital health information and traditional health information are seamlessly intertwined [29]. Parents with university education were found to be the least likely to use social media for health information before or after a health consultation, which is consistent with other studies [2]. This may reflect literacy or health literacy confidence. Parents who have higher levels of education may be more confident to seek health information, resulting in being able to ask pertinent questions and better understand the health information received during a health consultation. This allows the parent to leave the health consultation feeling satisfied with the information they have received [30].

Previous studies have sought to understand why parents use social media for general health information. Reasons have included social media’s information immediacy [20]; timely access despite geographical [21] or logistical [15] barriers; detailed, customized, and relevant information [20]; and perceived trustworthiness [31]. Parents view social media as being unbiased [20], aligning with their personal perspectives [21] and values [32], and providing insights to lived experience not available elsewhere [19,21].

Although parents’ use of social media before a health consultation is often to seek information about a health issue or to determine if treatment is needed, some of the reasons why parents may use social media for health information after a consultation raise questions about communication and health literacy. Almost half of all Australian adults read at a low level [33] and 60% have low levels of health literacy [34], with both of these factors potentially creating barriers to parents’ understanding of traditional health information. Social media health information is multimodal, combining personal stories, conversational text, videos, infographics [35], subtitles, and other design features that make it more inclusive for those with varying literacy levels [36]. The interactive and conversational nature of social media makes information more accessible, making it a preferable source for some parents [32]. Our nationally representative study shows that this is not only the experience of parents who are part of specific niche groups, as shown in the extant literature [14,16,18,19], but is true of the wider parenting experience.

Health information goes beyond the evidence-based information provided by health care professionals in consultations [2]. Parents seek emotional support [2] on social media and insights into how the health journey will impact their child, themselves, and their wider orbit. This information (which is often practical [32]) from other parents with lived experience is highly valued and sought after, allowing parents to feel more empowered and socially supported [37], thus increasing self-efficacy [16,38]. The democratized sharing of stories of lived experience is a unique feature of social media, which is also a strong motivator for parents who use social media for health information. Stories of lived experience allow parents to see what might lie ahead for their health journey, providing reassurance while also allowing them to allay uncertainties. It also allows parents to get health information beyond the clinical data, with practical tips and help to navigate the health system. Although these are only a few examples of information sought by parents based on lived experience, the power of stories for health communication has been long established. A scoping review by Dudley et al [39] found that narratives are appealing to audiences, stimulate emotions, make it easier to understand health and science information, improve the memory of information, and capture attention through suspense. Stories also “enable people to make sense of themselves, others, relationships, responsibilities, life changing circumstances, uncertainties, their social world, and possible futures” [40], all of which are heightened at emotionally vulnerable times such as when a child is unwell. Stories also make the parent more open to the messaging held within a story, whether that be evidence based or not, which is where social media starts to reveal its complexity as a health information source. Although stories on social media make health information easier for parents to understand, the use of social media for health information brings with it health literacy challenges unique to social media. For example, when using social media to seek health information, parents need to be able to distinguish evidence-based health information from low-quality information, a lot of which is delivered by stories, within conversations, or as part of a social information exchange. This requires a parent to be able to navigate the dynamic social context they are currently in; use high-level health literacy skills; and if required, research context and the original source of information, all simultaneously in real time. Rarely, if at all, has this combination of skills been required previously of parents when seeking health information, let alone at the level of sophistication that is often required by social media. As a result, the lack of the unique health literacy skills as required by social media often results in parents being ill-equipped to navigate the health information available on social media.

For health care professionals, it may be of value to consider how to integrate better provision of accessible evidence-based health information to parents [41] into their practice. By accepting the use of social media for health information as the “new normal,” clinicians can also facilitate frank conversations with parents [42] about reliable web-based information sources and offer high-quality information in more accessible forms, such as referring patients to videos formatted for viewing on mobile phones and social media content known to be evidence based or facilitating live question and answer sessions on Instagram or TikTok.

Finally, the impacts of using social media for health information will inevitably seep into other aspects of the health system, including public health. This can perhaps be seen most clearly with preventable childhood infectious diseases. With parents independently accessing non–evidence-based, emotively laden, and politically motivated health information [41], primary prevention gains may be lost (whether it be lowered rates of disease or the elimination of disease) with an increase in outbreaks of diseases, as seen overseas [43] such as the Disneyland measles outbreak in 2014 [8]. This led to public health units needing to invest in health promotion resources to highlight the importance of vaccination and fund programs to boost vaccination coverage to sustain herd immunity.

The scarcity of accessible evidence-based health information that meets parents’ information needs leaves parents vulnerable to finding low-quality health information when they turn to social media. Inclusive, accessible, and evidence-based health information urgently needs to be more readily available at all levels, from public health units down to in-consultation resources for health care professionals to guide conversations, as well as postconsultation resources for parents to take home. This will allow parents to consider evidence-based information in their own time, improve patient education, and reduce the reliance on non–evidence-based health information found on social media [44].

### Limitations

Inherent with any cross-sectional study design, responder bias is a confounding factor. Although measures were taken to limit the impact of responder bias, including having very broad inclusion criteria not related to the survey questions, as well as the stratification of data to the Australian Bureau of Statistics 2016 census data, the fact that the participants were from the research company’s preselected panel is a limitation.

Second, this survey required proficiency in English. Although the participants were stratified to be representative of the broader Australian population, not providing the survey in multiple languages limits representativeness in a multicultural society.

Third, the inclusion criteria stipulated that only parents with a social media account were to be included in this study. To access this survey, parents had to be able to access an internet connection; as such, we did not ask about their access to the internet as infrastructure was outside the scope of this study. This, however, did limit the study to only include those that have access to both the internet and social media.

Lastly, although cross-sectional studies cannot demonstrate causation, this study establishes a baseline for further research in this emerging area, which has substantial implications for clinical practice.

### Conclusion

With many Australian adults having low levels of health literacy, and almost half of all parents who used social media after a consultation reporting that the information from their health care professional was unclear, how evidence-based health information is delivered needs to be reconsidered to meet parents at their health literacy level. This could include resources that take a similar form to those found on social media that parents are already engaging with, such as those that are simplified, graphic, or video based. Public health units and, more broadly, the health system can support clinicians with their education of parents by providing inclusive health promotion communications and resources that are reliable and evidence based and meet parents at their health literacy level. Parents of patients could then be directed to quality resources, leading to health decisions that are informed and supported by evidence-based health information.

## Abbreviations

OR: odds ratio
PIS: participant information sheet
QOR: Quality Online Research
STROBE: Strengthening the Reporting of Observational Studies in Epidemiology
